# Preparation and characterization of polysaccharide - silica hybrid aerogels

**DOI:** 10.1038/s41598-019-52974-0

**Published:** 2019-11-11

**Authors:** Gabrijela Horvat, Milica Pantić, Željko Knez, Zoran Novak

**Affiliations:** University of Maribor, Faculty of Chemistry and Chemical Engineering, Smetanova 17, SI-2000 Maribor, Slovenia

**Keywords:** Implants, Organic-inorganic nanostructures

## Abstract

Hybrid aerogels based on polysaccharides - silica were prepared and characterized. Tetramethylorthosilicate (TMOS) was used as inorganic precursor and various polysaccharides (alginate, pectin, xanthan and guar) were used as organic precursors. TMOS was added to polysaccharide aqueous solutions, resulting in stable wet gels. There were no additional chemicals or cross-linkers in the process. Produced wet gels were dried under supercritical conditions with CO_2_ in order to preserve their structure. The nitrogen adsorption results were compared to pure polysaccharide aerogels, prepared in our previous research. It is shown, that the addition of silica to pectin, xanthan, alginate and guar significantly improved their structural properties, primarily seen in the drastic increase of the surface area. Guar-silica aerogels reached the highest surface area of 679 m^2^ g^−1^. The thermal properties, including thermal degradation and thermal conductivity were highly improved. Among the prepared hybrid aerogels, pectin-silica samples had the lowest thermal conductivity of 19 mWm^−1^ K^−1^.

## Introduction

Aerogels are materials with low density (0.004–0.500 gcm^−3^), large internal surface area and open pores, and are thus promising candidates for various advanced applications^1^. Silica aerogel, as an example, is prepared by the sol-gel process and has some useful properties, such as ultralow density and low thermal conductivity, even lower than that of free air. This is the result of its mesoporous structure, which allows decreasing the thermal conductivity of gaseous phase below that of air as a result of the Knudsen effect. The large-scale industrial application of silica aerogels, prepared from different silica precursors, such as Tetramethylortosilicate (TMOS) or Tetraethylortosilicate (TEOS), has been hampered by their poor mechanical properties^2^. On the other hand, bio-based aerogels, often prepared from various polysaccharides, are a new generation of aerogels developed in the past decade^3–6^. They provide better mechanical properties and can even achieve extreme low thermal conductivities, almost comparable to those of silica aerogels^7,8^. The reinforcement of silica aerogel with polysaccharide is therefore one way to improve its extreme fragility. By weaving a biopolymer through a nanomaterial, such as aerogel, the biopolymer will have both the physical and the chemical properties of the hybrid materials^9^.

Organic-inorganic materials are hybrid materials with organic and inorganic components, intimately mixed^10^. The inorganic phase can change the mechanical and thermal properties of pure organic materials and vice versa. Additionally, hybrid materials often exhibit new functionalities such as magnetic and electric properties, increased adsorption or better structural properties^11–16^. Hybrid materials are often prepared using the sol-gel process, which is also the process for the preparation of both inorganic and organic aerogels. The reason probably lies in the mild synthetic conditions, such as metallo-organic precursors, low processing temperature and the versatility of the colloidal state^10,16,17^. In most sol-gel processes, the inorganic framework is built after hydrolysis and condensation reactions. This also applies to silicon alkoxide, used in this study.

Silica nanocomposite materials are usually prepared through three steps. Initially, the alkoxide is mixed with water and hydrolysis takes place. Then the condensation reaction of silanols produces oligomers. The gel is formed after a cross-linking of the sol particles. To promote the last stage, it is usually necessary to introduce a catalyst, often an acid or alkali^17^.

The gelation of polysaccharide alone is usually trickier, since almost every polysaccharide has its own gelation route. However, in one of our recent papers, we proposed the preparation of various polysaccharide aerogels only with the addition of alcohol to polysaccharide solution^3^. It was reported^17^ that polysaccharides act also like good catalysts in the sol-gel process with silica, therefore the TMOS and polysaccharides could be a good combination for the formation of advanced composite materials.

Until now, only a few studies on preparing hybrid polysaccharide-silica aerogels have been reported. Most research has been done on cellulose – silica aerogels^11,18–21^ and chitosan – silica aerogels^22–24^. Alginate – silica aerogels were prepared in the form of a silica core, encapsulated by an alginate aerogel layer^25^. One of the latest studies in the field reports the one-pot synthesis of superinsulating pectin-silica aerogels with improved mechanical properties^26^.

To the best of our knowledge, there are no studies reported to date on the synthesis of silica-polysaccharide aerogels by the route described in this paper. Moreover, this is the first report on xanthan – silica and guar – silica aerogels. In this paper, the preparation of polysaccharide-silica aerogels is described using the sol–gel process in order to build an organic–inorganic network with improved thermal and structural properties.

## Materials and Methods

### Blank TMOS aerogel preparation

Tetramethylorthosilicate (TMOS) (≥99%, CAS: 681-84-5) was obtained from Sigma Aldrich, USA.

TMOS (as obtained) was poured into the 20 mL of a stirring ultra-pure water. Three aqueous solutions were prepared by adding 2, 4 and 6 mL of TMOS, respectively. Solutions were transferred to moulds and left for 24 h. After the gelation methanol was added on the top of a gel and replaced after 5 h.

### Blank polysaccharide aerogel preparation

High methoxyl pectin (hmP) (degree of esterification: 78%, CAS: 9000-69-5) was provided by Herbstreith & Fox, Germany. Alginic acid sodium salt (from brown algae, CAS: 9005-38-3), xanthan gum (CAS: 11138-66-2) and guar (CAS: 9000-30-0) were purchased from Sigma Aldrich, USA.

Blank polysaccharide aerogels were prepared as described in our previous paper^3^, by dissolving either 0.4 g or 0.8 g of hmP and 0.4 g of alginate, xanthan and guar in 20 mL of water, respectively. 10% wt of absolute ethanol was added and the solutions were placed to moulds, where additional ethanol was added on the top of the solution for gel to set.

### Hybrid aerogel preparation

#### Polysaccharide-silica gels

The preparation of hybrid gel started with the preparation of 2 wt% and 4 wt% pectin aqueous solutions and 2 wt% alginate, xanthan and guar aqueous solutions. Either 0.4 g or 0.8 g of hmP and 0.4 g of alginate, xanthan and guar was dissolved in 20 mL of water. pH of prepared pectin solutions were 3.23 and 3.12 for 2% and 4%, respectively and 7.55, 5.00 and 6.20 for alginate, xanthan and guar, respectively. The solutions were mixed until homogenization at 400 rpm and 25 °C for 1 h and then TMOS was added to each solution, respectively. The prepared polysaccharide-silica solutions were transferred to moulds and left until the gels were set. The parameters for all prepared samples are shown in Table 1.

**Table 1:** Composition of prepared samples.

Sample	Polysaccharide/g	TMOS/mL	Polysaccharide	TMOS
			wt%	wt%
Si2	0	2	0	100
Si4	0	4	0	100
Si6	0	6	0	100
hmP2-Si2	0.4	2	16	84
hmP2-Si4	0.4	4	9	91
hmP2-Si6	0.4	6	6	94
hmP4-Si2	0.8	2	33	67
Al2-Si6	0.4	6	6	94
Xa2-Si6	0.4	6	6	94
Gu2-Si6	0.4	6	6	94
hmP2	0.4	0	100	0
hmP4	0.8	0	100	0
Al2	0.4	0	100	0
Xa2	0.4	0	100	0
Gu2	0.4	0	100	0

### Supercritical drying of gels

Obtained wet gels were firstly aged in methanol for 5 h. Then the methanol was replaced for a fresh one and gels were aged until next day. Then the supercritical drying was performed in order to retain the structure of wet gels. The gels were transferred into a 500 mL autoclave and dried in the presence of supercritical carbon dioxide. The autoclave was preheated to 40 °C and then the system was slowly pressurized with CO_2_ up to 120 bars. The extraction of methanol from wet gels with supercritical CO_2_ was performed for 6 h. Then the system was slowly depressurized and cooled down to room temperature. Obtained aerogels were characterized afterwards.

### Characterization of aerogels

Surface and cross-sectional morphologies of the silica and silica-polysaccharide hybrid aerogels prepared in this study were observed by using field emission scanning electron microscopy (FE-SEM) (HRSEM, Helios Nanolab FEI 650). The samples were fractioned and then sputter-coated with gold particles (JEOL-JCF-1100E) and scanned at an accelerating voltage of 5 kV. The adsorption capacity, surface area, pore volume and pore size distribution were recorded on Micromeritics ASAP 2020 by nitrogen adsorption at 77 K. BET model was used for evaluation of surface area.. Thermogravimetric (TG) analysis was performed using a TGADSC1 Mettler Toledo in the temperature range of 30–600 °C at a heating rate of 10 °C min^−1^. Thermal conductivity of hybrid aerogels (diameter of sample: 6 mm, thickness: 0.5–1.0 mm) was measured on HPDSC1 at the melting point of indium at 156.6 °C. Analysis was performed under N_2_ atmosphere (50 mlmin^−1^) at 0.5 Kmin^−1^ heating rate. The method for measuring thermal conductivity on HPDSC1 is described in one of our previous papers^27^. Heat flow was measured by heating from 153 to 162 °C at 0.5 °C min^−1^. Purge gas was nitrogen with a gas flow of 50 ml min^−1^.

The slope *S* (left side of the melting peak) is determined by plotting the DSC curve against temperature^28^. This slope is essential for the determination of thermal conductivity according to Eq. 1:

1 $$S=\frac{\varphi }{\Delta T}$$

where *Φ* is the heat flow and *ΔT* is the difference between the temperature of the sample at time *t* and the melting point of the metal (*T*_*onset*_). For determination of thermal conductivity, we then used Eq. 2:

2 $$\lambda =\frac{\varphi }{\Delta T}\frac{h}{A}=S\frac{h}{A}$$

where *h* is the sample height, *A* is the cross-sectional area and *λ* is thermal conductivity in units (WK^−1^ m^−1^).

The experimental results are expressed as the mean ± standard deviation (SD) of three parallel experiments (n = 3).

True densities (ρ) of aerogels were measured by gas pycnometer (Micromeritics AccuPyc II 1340). Bulk densities were then determined as the ratio of mass to volume. The mass of the aerogel was determined by five-digit analytical balance (Mettler Toledo) and the volume was determined by measuring the dimensions of a cubical shaped aerogel. Porosity was determined as the ratio between bulk and true density by Eq. 3

3 $$\varepsilon ( \% )=(1-\frac{{\rho }_{{\boldsymbol{B}}}}{{\rho }_{T}})\cdot 100$$

where ${\rho }_{{\boldsymbol{B}}}$ is the bulk density of the aerogel, ${\rho }_{T}$, is the true density of aerogel.

## Results

**Figure 1:**
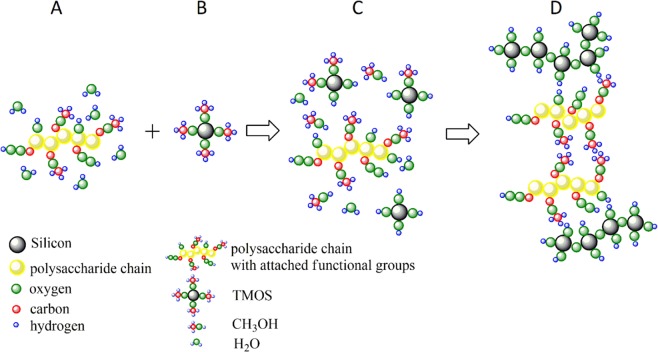
A hypothesis of gel formation between polysaccharide and TMOS for producing hybrids. (**A**) Polysaccharide aqueous solution. (**B**) Silica precursor. (**C**) Bonding between polysaccharide and silica (**D**) final hybrid wet gel.

Blank TMOS aerogels were prepared as described in the materials and methods section. A hypothetical model of the gel formation from polysaccharide and silica is proposed in Fig. 1 and by Eqs 4 and 5. It is based on the hydrolysis reactions of TMOS (Eq. 4)^29^, and hydrophobic interactions in the molecule of polysaccharide, presented in the Fig. 1 as A + B → C. Final formation of a gel occurs due to the weak interactions between silica and polysaccharide based on H-bonds (Eq. 5), presented in the Fig. 1 as C → D.

4 $$\begin{array}{c}\mathrm{nPolysaccharide} \mbox{-} ({{\rm{COOCH}}}_{3})+4{{\rm{H}}}_{2}{\rm{O}}+{\rm{nSi}}{({{\rm{OCH}}}_{3})}_{4}\to ({\rm{OH}})4+4{{\rm{CH}}}_{3}{\rm{OH}}\\ \,+\mathrm{nPolysaccharide} \mbox{-} ({{\rm{COOCH}}}_{3})\end{array}$$

5 $$\begin{array}{c}{\rm{nSi}}{({\rm{OH}})}_{4}+\mathrm{nPolysaccharide} \mbox{-} ({{\rm{COOCH}}}_{3})+4{{\rm{CH}}}_{3}\to {(-O-\mathrm{Si}({{\rm{OH}}}_{2}))}_{{\rm{n}}}\\ \, \mbox{-} \mathrm{polysaccharide} \mbox{-} ({{\rm{COOCH}}}_{3}){({}_{3}{\rm{HCOOC}} \mbox{-} {\rm{polysaccharide}})}_{{\rm{n}}} \mbox{-} (\,-O-\mathrm{Si}({{\rm{OH}}}_{2})\end{array}$$

**Figure 2:**
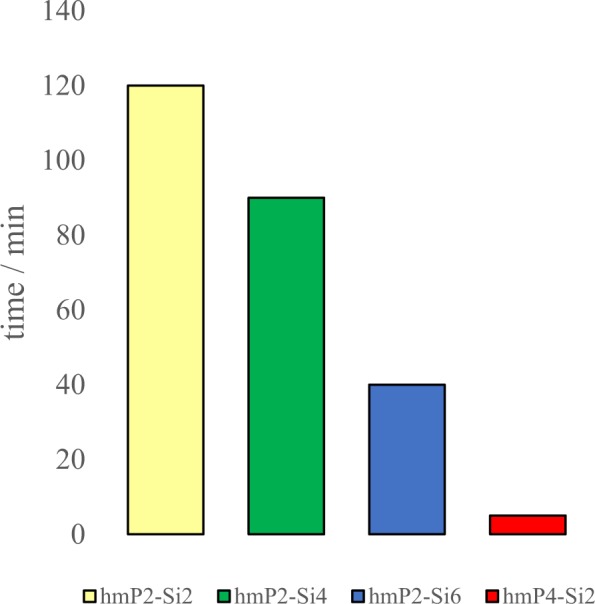
Gelation time of hmP-Si hybrids.

Firstly, only hmP-Si hybrids were prepared in order to investigate, wheather the hmP would form a gel with the addition of TMOS only. Later, the impact of the concentration of the hmP aqueous solution and amount of TMOS were investigated. Gelation times for all hmP-Si hybrid gels were monitored and are presented in Fig. 2.

**Figure 3:**
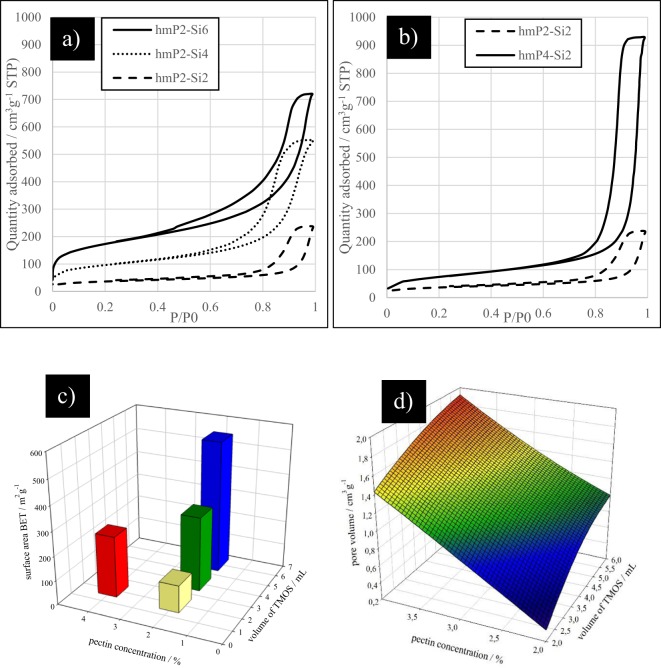
Nitrogen adsorption: (**a**) adsorption isotherms of hybrid aerogels for different TMOS volumes, (**b**) adsorption isotherms of hybrid aerogels for different pectin concentrations, (**c**) surface area of hmP-Si hybrid aerogels and (**d**) the pore volume of hmP-Si hybrid aerogels.

Nitrogen adsorption results are gathered in the Fig. 3. Figure 3a shows the difference between the adsorption isotherms when the volume of TMOS increased, while hmP concentration remained the same. In the Fig. 3b it is presented, that when the hmP concentration doubles, the adsorption capacity became 4 times higher.

Figure 3c shows the difference in the surface area of hybrid aerogels, when the volume of TMOS and the concentration of hmP are increased. When the hmP concentration is doubled from 2 wt% to 4 wt%, the surface area increases from 112 m^2^ g^−1^ to 251 m^2^ g^−1^. However, when the volume of TMOS is increased from 2 mL to 4 mL and 6 mL, the surface area increases from 112 m^2^ g^−1^ to 306 m^2^ g^−1^ and 543 m^2^ g^−1^, respectively. It can be seen that pore volume increases upon increasing the volume of TMOS and increasing the polysaccharide concentration (Fig. 3d). Four-fold higher pore volume is reached then by tripling the volume of TMOS. The red/orange area in this plot shows theoretical results which could not be obtained at room temperatures, because of the rapid gel setting.

**Figure 4:**
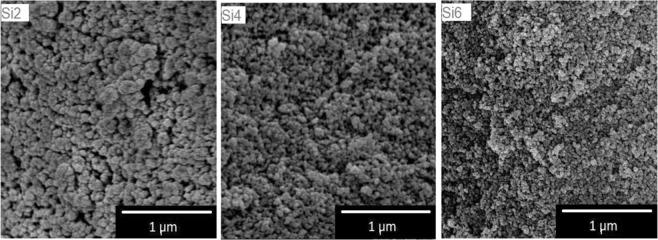
FE-SEM images of blank silica aerogels.

**Figure 5:**
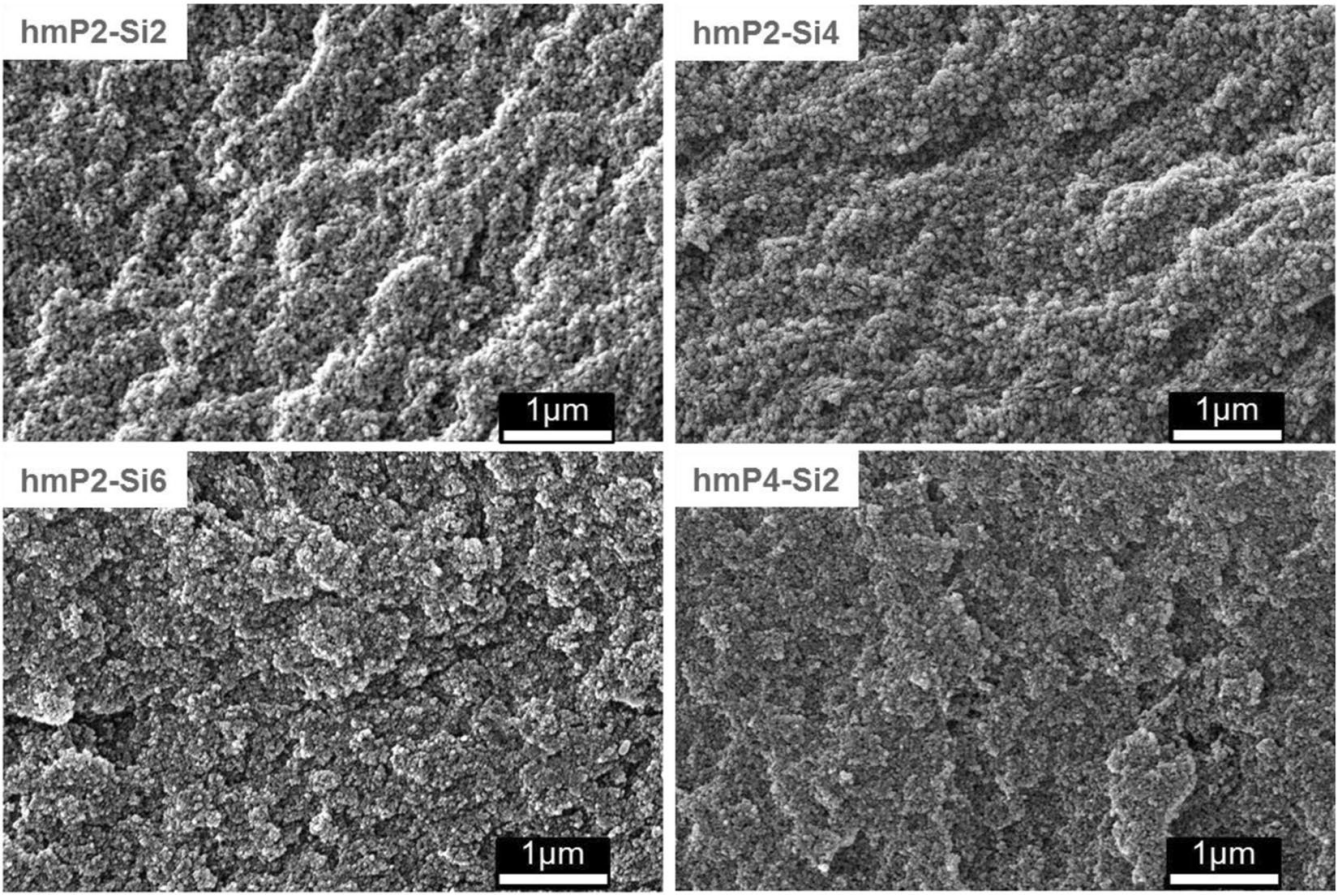
FE-SEM images of hmP-Si hybrid aerogels.

Figure 4 presents the FE-SEM scans of blank silica aerogels. The difference between samples is notable. The grape-like structure is typical for all three samples, however by increasing the amount of TMOS in water, those clusters become smaller. FE-SEM pictures of hybrid pectin aerogels are presented in Fig. 5. It can be seen that the structure of all of the hybrid materials is highly compact with almost no large voids present in the structure.

Later, hybrid aerogels from other polysaccharide sources and TMOS were prepared. Based on the results for hmP-Si aerogels, 2 wt% polysaccharide solution and 6 mL of TMOS were used for the preparation of Al2-Si6, Xa2-Si6 and Gu2-Si6 hybrid aerogels. Table 2 shows the results of nitrogen adsorption. The results were rather surprising, especially for Gu2-Si6 aerogels. The surface area of this sample, 679 m^2^ g^−1^, was the highest; however, in our previously published paper^3^ we reported the lowest surface area (111 m^2^ g^−1^) for blank guar aerogels, compared to other polysaccharide aerogels. Similarly, the surface area of Al2-Si6 aerogels is much improved compared to blank alginate aerogels (147 m^2^ g^−1^)^3^. It is well known that aerogels are materials with high porosities and low densities. The porosity of the prepared materials was determined as the ratio between bulk and true density of the material, as shown by Eq. 1 and is presented in the Table 2.

**Table 2:** Nitrogen adsorption.

Sample	Surface area BET m^2^ g^−1^	Pore volume BET cm^3^ g^−1^	Pore volume from density cm^3^ g^−1^	Bulk density gcm^−3^	True density gcm^−3^	Porosity/%
Si6	973 ± 12	3.6	3.9	0.06	1.7	96.5 ± 0.9
hmP2-Si6	543 ± 15	1.1	1.2	0.10	1.8	94.7 ± 1.3
Al2-Si6	556 ± 17	0.4	0.6	0.13	2.0	93.5 ± 1.9
Xa2-Si6	621 ± 19	0.2	0.7	0.13	1.7	92.5 ± 0.9
Gu2-Si6	679 ± 9	0.5	0.6	0.13	1.7	92.3 ± 2.3
hmP2	354 ± 12	1.9	2.1	0.11	2.6	93.8 ± 0.6
hmP4	384 ± 5	2.1	2.2	0.10	2.2	95.5 ± 1.2
Al2	135 ± 10	0.3	0.8	0.18	2.2	91.8 ± 0.4
Xa2	342 ± 11	1.5	1.8	0.17	2.0	91.5 ± 0.5
Gu2	103 ± 15	0.4	0.9	0.29	2.2	86.9 ± 0.2

**Figure 6:**
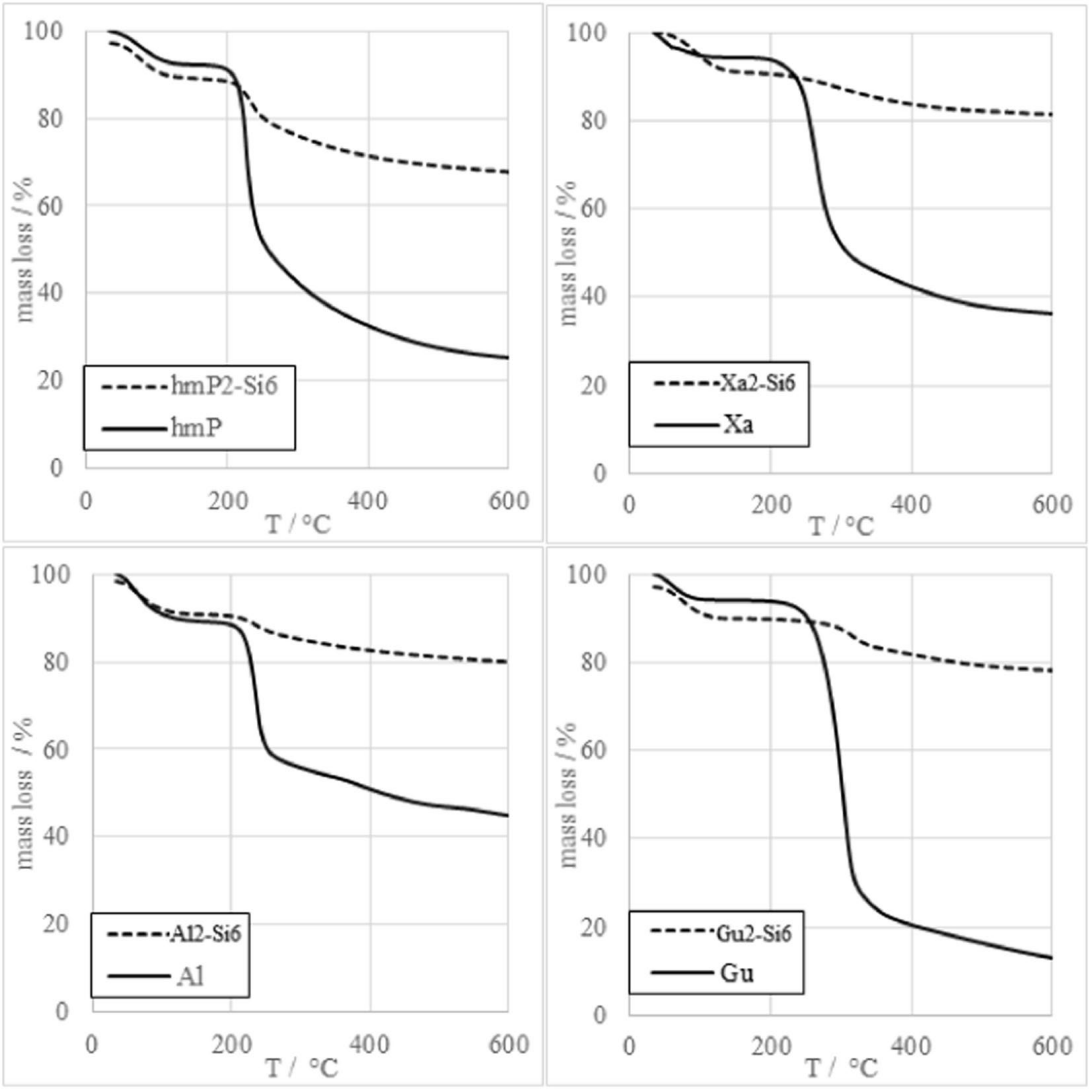
TG analysis of hybrid aerogels.

TG analysis was used to determine the thermal stability of hybrid aerogels. We compared their degradation to the degradation of blank polysaccharide samples, published by previously by our group^3^. As can be seen in Fig. 6a–d, hybrid aerogels are much more stable and resistant to thermal degradation than their parent polysaccharide aerogels.

**Figure 7:**
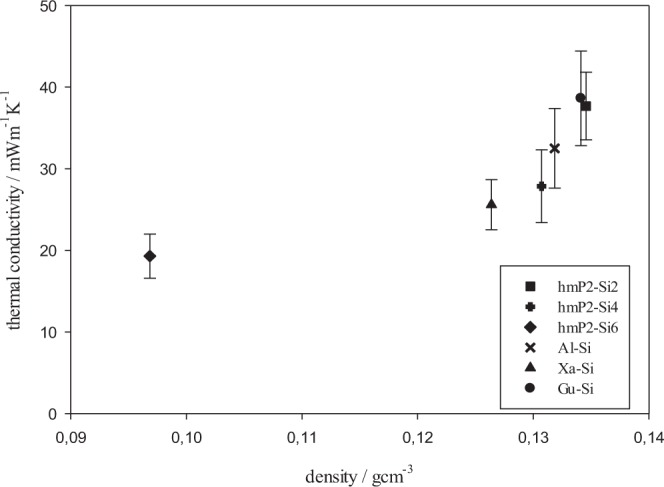
Thermal conductivity as the function of density.

Thermal conductivity was determined by the DSC method described in^27^. It is shown in Fig. 7 that thermal conductivity increases almost exponentially with higher aerogel density. Already reported thermal conductivity of pectin – silica hybrid aerogel (19 mWm^−1^ K^−1^) places this hybrid material among the best thermal insulators^26^. The thermal conductivity of hmP2-Si6 hybrid aerogel prepared in this study therefore falls into the region of thermal superinsulators.

## Discussion

Xanthan and guar gum have a linear backbone to which short chains are attached. Therefore, they are classed as graft-polymers and alginate and pectin are block copolymers. Xanthan is capable of jellifying an aqueous solution^17^. Previous findings published by our research group^3,30^ show that polysaccharide can form stable gels with the addition of alcohol to a polysaccharide aqueous solution; therefore, without any additional cross-linker. Ethanol or methanol induce the gel formation in various polysaccharides, including pectin, xanthan, alginate and guar^31^, as the result of hydrophobic interactions. After the hydrolysis of TMOS, methanol is formed^32^; therefore, it was expected that the polysaccharides can also form a gel with the addition of TMOS to their aqueous solution. The addition of extra alcohol to the system is unnecessary, since the alcohol released during the reaction is sufficient to convert the initially biphasic system into a homogeneous one^33^.

Alkoxy groups in TMOS are usually hydrolysed when the compounds are placed in an aqueous environment, particularly in the presence of acid or basic catalysts, to generate silanol groups^34^. In the polysaccharide - H_2_O - TMOS system, the addition of the catalyst is not necessary, since the carboxylic groups in polysaccharides increase the rate of the hydrolysis of TMOS. The effect of polysaccharides on the sol-gel processes was most pronounced at pH’s near neutral. To accelerate the sol-gel processes, it is necessary to add a catalyst, for example, an acid. Therefore, it was concluded that they exert a catalytic effect on the sol-gel processes^17^. Under acidic conditions, however, the hydrolysis is slower compared to base-catalysed hydrolysis^35^. The silica therefore tends to form linear molecules that are occasionally cross-linked^34^. These molecular chains entangle and form additional branches resulting in gelation, forming an interpenetrating polymer network.

Gelation times were highly dependent on the hmP and Si ratio. Gel set faster when a higher hmP concentration (4 wt%) was used, as a result of the higher amount of COO^-^ groups to promote TMOS hydrolysis, as well as the higher amount of COCH_3_ groups to promote hydrophobic interactions. Because of the almost immediate gelation of hmP4-Si2, the addition of a higher amount of TMOS to 4 wt% hmP solution is not possible at 25 °C. Additionally, gelation time decreased almost linearly as the volume of the TMOS increased, because of the higher gelation rate between silanol groups and higher amount of released methanol after the hydrolysis of TMOS, hence stronger hydrophobic interactions between polysaccharide chains.

As the volume of TMOS increases, macroporous region decreases (Fig. 3a). Apparently, there are more mesopores and the adsorption capacity increases exponentially. It is interesting to note that when the hmP concentration doubled, the adsorption capacity became 4 times higher (Fig. 3b). This is probably caused by the highly reduced macroporus region. This could be the proof of silica filling the macropores of polysaccharide aerogels. The addition of a larger amount of TMOS is not possible because of the rapid gelation at room temperature. From Fig. 3a,b it can be seen that polysaccharide content plays a much more important role in increasing adsorption capacity, compared to increasing the volume of TMOS.

When the hmP concentration is doubled, the surface area doubles, and when the volume of TMOS is doubled, the surface area triples (Fig. 3c). Therefore, the amount of TMOS has a higher effect on the surface area compared to polysaccharide concentration, which is expected as a result of the much higher surface area of blank silica aerogel compared to that of blank pectin aerogel. Surface area of hmP-Si is rather low, compared to our previously published paper of blank hmP aerogels. Reported surface area for blank 4% hmP aerogel is 384 m^2^ g^−13^. Polysaccharide concentration has a higher impact on pore volume, but TMOS influences mostly on the surface area. As can be seen from Fig. 3d, pore volume (BJH) increases more than four-fold when the polysaccharide concentration is doubled. Silica is most likely filling the macropores regions in polysaccharide aerogel, but also higher concentration of polysaccharide aerogel leads to more compact structure and thus lower macropore and higher mesopore volumes.

Surface areas of 2% guar, alginate, xanthan and guar aerogels are slightly lower from our previously published results on 4% polysaccharide aerogels^3^. This is expected due to the larger macropore volume. However, silica reference sample has similar surface area to what is reported in the literature, this is around 800–1000 m^2^ g^−1^ ^36^. The specific surface area of all hybrid samples, prepared from 2% wt polysaccharide solutions with 6 mL of TMOS increased (Table 2). The highest increment was observed for guar and alginate aerogels. This result is a confirmation of the presence of nanostructured silica aerogel phase in the pores of polysaccharide aerogels. Since blank alginate and guar aerogels have the highest volume of macropores, there is more space for silica and thus higher surface area of those hybrid materials.

Addition of silica indeed improves the thermal stability of samples. The highest difference was observed in Gu2-Si6 aerogels, where guar aerogels degraded to around 30% of their initial weight at 330 °C and hybrid aerogel degraded by only 16% at the same temperature. At 600 °C, Al2-Si6, Gu2-Si6 and Xa2-Si6 degraded by about 20%. Only hmP2-Si6 degradation is higher, degrading by about 35% at 600 °C. Other groups reported the stability of pectin-silica hybrids up to 250 °C^26^.

Blank silica aerogel network is formed of nanometric silica beads with no observable macropores (Fig. 4). Higher concentration of silica leads to networks with smaller beads. Networks with smaller beads should have larger surface areas. This was proven with nitrogen adsorption. Determined surface area was 857 m^2^ g^−1^, 878 m^2^ g^−1^ and 973 m^2^ g^−1^ for Si2, Si4 and Si6, respectively. SEM images of hybrid hmP-Si aerogels are in agreement with other literature, with the very compact microstructure and grape-like structure^26^.

Prepared hybrids from different polysaccharides and TMOS had high surface areas. The most notable difference was observed by Gu2-Si6 aerogel, where the surface area increased from 103 m^2^ g^−1^ up to 679 m^2^ g^−1^ compared to the blank guar aerogel. This is most likely due to higher macropore volume in guar aerogels, which are filed with silica.

Thermal conductivity was determined by the DSC method described in^27^. The accuracy of this method is about ±10% to ±20%^28^. It is shown in Fig. 7 that thermal conductivity increases almost exponentially with higher aerogel density. The density plays a major role in producing thermally superinsulating materials. The alignment of the aerogel structure could possibly be the one of the major influencers on the thermal conductivity^37^. Silica aerogels have lowest thermal conductivities, even as low as 15 mWm^−1^ K^−1^ ^36^ but they are likely brittle^18^. The addition of polysaccharide may benefit their mechanical properties. Thermal conductivity of pure polysaccharide aerogels is higher, due to the presence of macropores. Since silica is filling those macropores, the thermal conductivity of hybrid aerogels is lower than that of pure polysaccharide aerogels, but higher of pure silica aerogels. Already reported thermal conductivity of pectin – silica hybrid aerogel (14–17 mWm^−1^ K^−1^) places this hybrid material among the best thermal insulators^26^. Pectin-silica aerogels have lower thermal conductivities and hence better insulation properties than other hybrid aerogels i.e. silica, reinforced with cellulose^7,38^, isocyanate^39^ and epoxy^40^. The thermal conductivity of hmP2-Si6 hybrid aerogel prepared in this study therefore falls into the region of thermal superinsulators. For all other samples, i.e. hmP2-Si2 and hmP2-Si4, Al2-Si6, Xa2-Si6 and Gu2-Si6, the superinsulation is not met. The densities of those hybrids are higher, which results in higher phonon conduction through the solid backbone and finally the higher thermal conductivity. Additionally, there may still be some remaining macropores, that are not filled with silica.

## Conclusions

Polysaccharide-silica aerogels were prepared by a new approach at room temperature and subsequent supercritical drying. Hybrid gels were set only by mixing polysaccharide solutions with TMOS without the additional cross-linkers or chemicals. The prepared samples showed useful structural properties, including highly increased surface area compared to pure polysaccharide aerogels. Hybrid aerogels show high thermal stability and low thermal conductivity. This simple method of mixing polysaccharide solution and TMOS is therefore an interesting and novel procedure for the production of polysaccharide-silica aerogels, and gives a new perspective and possibilities for the study of such advanced hybrid materials.
